# Collusion-Aware Privacy-Preserving Range Query in Tiered Wireless Sensor Networks^†^

**DOI:** 10.3390/s141223905

**Published:** 2014-12-11

**Authors:** Xiaoying Zhang, Lei Dong, Hui Peng, Hong Chen, Suyun Zhao, Cuiping Li

**Affiliations:** Key Laboratory of Data Engineering and Knowledge Engineering of Ministry of Education, School of Information, Renmin University of China, Beijing 100872, China; E-Mails: xiaoyingzhang1987@126.com (X.Z.); donglei1001@163.com (L.D.); huipeng@ruc.edu.cn (H.P.); zhao.suyun@yahoo.com (S.Z.); cuiping_li@263.net (C.L.)

**Keywords:** Internet of Things, wireless sensor networks, privacy preservation, integrity verification, range query

## Abstract

Wireless sensor networks (WSNs) are indispensable building blocks for the Internet of Things (IoT). With the development of WSNs, privacy issues have drawn more attention. Existing work on the privacy-preserving range query mainly focuses on privacy preservation and integrity verification in two-tiered WSNs in the case of compromised master nodes, but neglects the damage of node collusion. In this paper, we propose a series of collusion-aware privacy-preserving range query protocols in two-tiered WSNs. To the best of our knowledge, this paper is the first to consider collusion attacks for a range query in tiered WSNs while fulfilling the preservation of privacy and integrity. To preserve the privacy of data and queries, we propose a novel encoding scheme to conceal sensitive information. To preserve the integrity of the results, we present a verification scheme using the correlation among data. In addition, two schemes are further presented to improve result accuracy and reduce communication cost. Finally, theoretical analysis and experimental results confirm the efficiency, accuracy and privacy of our proposals.

## 1. Introduction

As indispensable building blocks for the Internet of Things (IoT), wireless sensor networks (WSNs) have been widely used in many applications, such as smart home, e-health and environment monitoring. In these applications, range query is an important type of query in WSNs, which aims at seeking all data falling into an attribute range specified by users. However, due to the openness and non-supervision of WSNs, privacy problems have been exposed when data are collected, transmitted or analyzed. For instance, in the field of e-health, wearable or fixed medical sensors are used to monitor patients' physical parameters (e.g., blood pressure) and respond to an emergency rapidly when needed. During the process of data collection, transmission or analysis, information of patients may be overheard by an adversary, such that patient privacy is compromised. Therefore, privacy-preserving range query processing is especially urgent.

**Figure 1.**
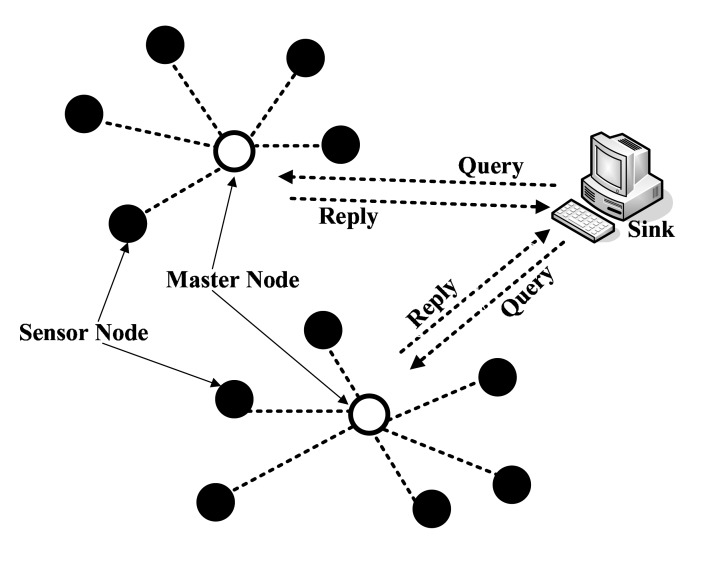
The architecture of two-tiered wireless sensor networks.

Due to resource savings, rapid response and high scalability, a two-tiered architecture [1] is adopted in the existing work [2–9] on privacy-preserving range queries. Figure 1 shows a typical two-tiered WSN, which consists of resource-limited sensor nodes in the lower tier and resource-rich master nodes, also called storage nodes, in the upper tier. Data are gathered by sensor nodes and transmitted to associated master nodes, and then, master nodes calculate and return results to the sink. Since WSNs are often deployed in hostile and unpredictable environments, the two-tiered architecture brings in serious security challenges. On the one hand, master nodes are untrustworthy and curious about data stored in themselves, so original data should be concealed before being submitted to master nodes. What is worse, the compromised master node may reply with fake or incomplete results. On the other hand, master nodes are required to perform queries efficiently and correctly, which is hindered if data are encrypted. As a result, it is challenging to preserve privacy and at the same time achieve efficient performance and correct results.

Previous studies [2–9] only focused on privacy preservation and integrity verification when the master node is compromised, without the consideration of collusion attacks. Besides, these studies preserve privacy and integrity at the detriment of efficiency and accuracy. To address the problem, we investigate a secure range query in two-tiered WSNs with the following contributions:

- We first propose a privacy-preserving range query protocol (PRQ) in two-tiered WSNs. In PRQ, we design a novel encoding scheme to preserve the privacy of data and queries and a verification scheme to check the result integrity. The encoding scheme represents data and queries by the special code, while the verification scheme discovers wrong results based on the ordinal relation of data.
- We then present a series of collusion-aware privacy-preserving range query protocols (CPRQ) based on PRQ. To the best of our knowledge, this paper is the first to fulfill the need for resistance to collusion attacks for a range query in tiered WSNs.
- We further present two schemes for improvements. The first scheme reduces communication cost by compressing the codes of the data. The second scheme improves result accuracy by denoting the query range as multiple codes.
- Theoretical analysis and experimental results indicate that our proposals can reduce the communication cost and accomplish more accurate results while preserving privacy and integrity.

The rest of the paper is organized as follows. Section 2 summarizes related work. Models and the problem statement are described in Section 3. Section 4 introduces preliminary knowledge. Then, the PRQ protocol and a series of CPRQ protocols are elaborated in Sections 5 and 6, respectively. These protocols are further improved in Section 7 and analyzed theoretically in Section 8. Section 9 evaluates our proposals using thorough experiments. We conclude this paper in Section 10.

## 2. Related Work

In recent years, privacy issues of WSNs have drawn increasing concerns [10]. The work in [11–22] and the work in [23–27] study the privacy preservation in data aggregation and the top-*k* query, respectively. However, our paper focuses on the range query. Privacy-preserving range query in two-tiered WSNs has been explored in [2–9].

The work in [2] is a milestone in the privacy-preserving range query. It uses the bucketing technique to divide the data domain into multiple disjoint buckets and represents data and queries by bucket IDs. If there are no data falling into a certain bucket, an encoding number is created for this bucket. After receiving a range query, storage nodes return satisfactory encrypted data and all encoding numbers. The bucketing technique protects privacy, while the encoding number verifies the integrity of the results. Nevertheless, it enables adversaries to obtain a reasonable estimation of the data and queries according to the range of the bucket and generates false positives in the results.

The work in [3] investigates the spatial and temporal relation among data. The major idea is similar to [2]. The difference is that it verifies the integrity by the bitmap instead of the encoding number. It cannot avoid false positives, and produces more communication. To reduce communication cost, [4] presents a probabilistic spatial-temporal crosscheck scheme, which fails to detect the compromised storage node absolutely.

SafeQ [5,6] adopts the prefix membership verification scheme [28,29] to ensure privacy and constructs the neighborhood chain to protect integrity. In addition, [6] establishes Merkle hash trees to examine the results. The disadvantage is that SafeQ needs more computation and communication.

The work in [7] proposes a privacy-preserving range query protocol on the basis of the order-preserving function, the permutation function and the *d*-disjunct matrix. The order-preserving function is used to process the query. The permutation function and the *d*-disjunct matrix are used to encrypt data and get the exact frequency of any value. It requires extra space to store matrices and cannot verify integrity.

Our previous paper [9] also focuses on the problem of the privacy-preserving range query in tiered WSNs. This paper is an extended version of [9] and is the first to take destructive collusion attacks into account for range query in tiered WSNs. Although [30] proposes a privacy-preserving location proof updating the system towards collusion resistance, it only protects location privacy in location-sensitive applications and is not suitable for privacy preservation of data and queries for the range query in WSNs. More protocols, theorems, improvements, theoretical analysis and experimental results are added in this paper.

## 3. Models and Problem Statement

### 3.1. Network Model

As illustrated in Figure 1, a two-tiered WSN is composed of three types of nodes: many sensor nodes, a few master nodes and a single sink. Sensor nodes have limited storage, computation, bandwidth and energy, while master nodes have strong capabilities and abundant resources as the sink. At the beginning of the network, system parameters are preloaded into sensor nodes. During the network lifetime, sensor nodes collect data (e.g., temperature and humidity) from their surroundings with a fixed frequency and then periodically transmit data to their master nodes. Master nodes store the data of their affiliated sensor nodes. Once they receive a range query from the sink, master nodes immediately search for required data and transmit the results to the sink. The network is considered to be separated into some non-overlapping subnetworks, and each subnetwork comprises a master node and several sensor nodes. Suppose all sensor nodes are synchronized, so that they keep consistent in epochs.

Compared with the traditional sensor network without master nodes, three major advantages of this architecture benefit the development of WSNs. First, resources are saved for sensor nodes. Master nodes store a large number of data and process complicated calculations, which cuts down the cost of storage and computation for sensor nodes and prolongs the network lifetime. Second, it improves the speed of the query response. Master nodes handle queries locally and send answers to the sink directly. Finally, network scalability is enhanced. The network is divided into subnetworks based on master nodes, and these subnetworks may be heterogeneous and run independently. As described in Section 1, the two-tiered architecture brings security challenges. More details will be given in the next subsection.

### 3.2. Adversary Model

There are two assumptions in our adversary model: (1) the sink is reliable, as assumed in [2–9]; otherwise, the entire network is trustless, since the sink can fabricate results; and (2) the number of compromised sensor nodes and master nodes is limited; if not, the network is disabled.

An adversary generally tries to eavesdrop on the sensitive data of the network through the wireless link layer, which violates data privacy. Moreover, once a node is compromised by the adversary, it will submit the wrong data, which breaches result integrity. Three cases against result integrity are discussed in the following:

- Compromising a sensor node: If a sensor node is compromised, the adversary can access its data and manipulate it to send incorrect data to its master node. For a certain range query, it has a slight and negligible effect on the final result. It is difficult to overcome this problem unless the hardware progresses.
- Compromising a master node: Storing lots of data, master nodes easily become the target of attacks. If a master node is compromised, it has a great impact on the network security. On the one hand, the adversary is enabled to obtain sensitive data stored in this master node. On the other hand, the master node dominated by the adversary may deliberately inject fake data or delete partial data, so that the sink receives incorrect results.
- Node collusion: If collusion occurs among sensor nodes, the influence on the network security is limited, as mentioned in the first case. However, if sensor nodes collude with their master nodes, all data stored in master nodes may be disclosed, and the damage to the whole network may be destructive.

It is clear that compromising the master node causes graver threats than the sensor node. Hence, our paper first pays attention to solving the problem of compromising master nodes in Section 5, and then enhances network security against collusion attacks in Section 6.

### 3.3. Problem Statement

A good privacy-preserving range query protocol should fulfill the following requirements.

- Data privacy: Since data are private and sensitive, all data should not be obtained by master nodes. Furthermore, data should be only known by their owner and the sink.
- Query privacy: The query range implicitly reveals the interest of users. Adversaries have the capability to infer the user's preference from the captured query range. Consequently, the query range should be only known by the sink.
- Result integrity: (1) All data in the results should be authentic, *i.e.*, forged data are not permitted; and (2) the result should include all data satisfying the query, *i.e.*, an incomplete result is not acceptable. At the least, the protocol should entitle the sink to detect actions of injecting fake data and removing legitimate data. Network security is reinforced by result integrity.
- Efficiency: Energy is the bottleneck of network lifetime. The work in [31] shows that communication consumes more energy than other processing in sensor nodes. Therefore, the less the communication cost, the higher the efficiency.
- Accuracy: Only the satisfactory data should exist in the result, and incorrect data should be excluded.

## 4. Preliminaries

The Bloom filter [32] is a space-efficient probabilistic data structure, which uses hash functions to support the membership test of a set. Although the Bloom filter may generate false positives, the benefit of space savings outweighs this drawback. In addition, hash functions can encode data. We adopt the Bloom filter to preserve privacy while testing membership in Section 5. In this section, we first briefly review the standard Bloom filter.

**Figure 2.**
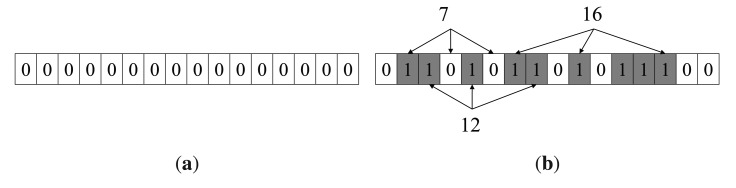
An example of the Bloom filter. (**a**) Initial Bloom filter; (**b**) check if 7, 12 and 16 are in the set {11,12,13,14,15}.

Assume that a Bloom filter consists of two parts: an array of *m* bits initialized to 0 and *k* independent one-way hash functions *h_i_*, …,*h_k_* mapping elements in the universe to a random number uniformly over the range {1, 2, …,*m*}. Given a set *S* = {*x_i_*|1 ≤ *i* ≤ *n*}, add an element *x* into *S*, just by setting all *h_j_*(*x*)-th (1 ≤ *j* ≤ *k*) bits to 1. Thus, the Bloom filter of *S* is constructed by setting all *h_j_*(*x_i_*)-th (1 ≤ *i* ≤ *n*, 1 ≤ *j* ≤ *k*) bits to 1. To examine if an element *y* is in *S*, check whether all of the *h_j_*(*y*)-th (1 ≤ *j* ≤ *k*) bits are set to 1. If not, *y* is definitely not in *S*. Otherwise, *y* may be in *S*. Either *y* is really in *S* or the *k* bits have been set to 1 by chance during the insertion of other elements in *S*, which is called a false positive. Figure 2 is an example of the Bloom filter. 0110101101011100 is the Bloom filter of the set *S* = {11,12,13,14,15}. Examine if *S* contains 7, 12 and 16, which are hashed to bits (2, 4, 6), 〈3, 5, 8〉 and 〈7,10,14〉, respectively. It is found that 7 is definitely not in *S*; 12 and 16 are considered to be in *S*, although 16 is actually not in *S*.

The probability of false positives is already given in [33]. It should be noticed that the probability of a false positive in [33] is different from that in this paper, because of the different properties of hash functions. False positives are allowable, as long as their probability is sufficiently low. In spite of efficient insertion and testing, the deletion of an element is troublesome. Because setting the related bits to 0 may result in removing any other elements hashed to those bits, this is called a false negative. False negatives are not permitted.

## 5. Privacy-Preserving Range Query

In this section, we propose a privacy-preserving range query protocol (PRQ). The protocol is divided into four steps: system initialization, privacy preservation, membership test and integrity verification. At the beginning, system parameters are initialized. Both nodes' data and users' queries are encoded to hide private information before being sent to master nodes. Then, master nodes search for matching data upon codes. Finally, result integrity is verified by the sink. More details are elaborated in the following. Table 1 summarizes the notation used in this paper.

### 5.1. System Initialization

Without loss of generality, let the data domain be a set of positive integers and *H* = {*h*_1_, *h*_2_, …} be a set of independent and randomly distributed hash functions, satisfying: for each datum *d*, there is *h_i_*(*d*) ≠ *h_j_*(*d*) (*i* ≠ *j*). The sink selects *k* different hash functions *h*_1_,…, *h_k_* from *H*. At the beginning of the network, *h*_1_,…, *h_k_* and *k_i_*_,0_ are preloaded in each sensor node *s_i_*, where *k_i_*,_0_ is a seed key and is only shared between the sensor node *s_i_* and the sink. *s_i_* encrypts its data by *k_i,t_*, which is the secret key of *s_i_* at epoch *t*. Let *k_i,t_* = *hash*(*k_i,t_*_–1_), and erase *k_i,t_*_–1_ at epoch *t*.

### 5.2. Privacy Preservation

As discussed in Section 3, data collected by sensor nodes and queries issued by users are private. Moreover, master nodes are unreliable and curious about private information. It is necessary to protect data and queries. In this subsection, a special encoding scheme is devised to preserve privacy by virtue of the excellent property of the Bloom filter.

We first consider privacy preservation of data. Assume the sensor node *si* gathers data *d*_1_, *d*_2_, …, *d_θ_* at epoch *t*. For each datum, construct a special code of *m* bits initialized to 0 by rules: map the datum to *k* positions by hash functions *h*_1_,…, *h_k_*, and set these positions of the code to 1. The encoding scheme is similar to the construction of the Bloom filter of a set with an element, so we call the special code *BFcode*. The message that the sensor node *s_i_* sends to its master node *M* is:

$$\begin{array}{c} s_{i}\to M:i,t,\{E(d_{0}\|0),E(d_{1}\|1),\ldots ,E(d_{\theta }\|\theta ),E(d_{\theta +1}\|\theta +1)\}\\ \left\{BF_{d_{0}},BF_{d_{1}},\ldots ,BF_{d_{\theta }},BF_{d_{\theta +1}}\right\} \end{array}$$

where *θ* is the number of data, *d_j_*(1 < *j* < *θ*) is the *j*-th data gathered by *s_i_* at epoch *t*, which is sorted in ascending order, *i.e., d*_1_ ≤ *d*_2_ ≤ … ≤ *d_θ_, d*_0_ and *d*_*θ*+1_ are system parameters for result verification, *d_j_*‖*j* is a combination of *d_j_* and *j*, also useful for checking results, *E*() denotes an encryption function using the secret key *k_i,t_*, and *BF_d_j__* represents the *BFcode* of *d_j_*. Without the right key, master nodes cannot decrypt correct data. Without the right hash functions, *BF_d_j__* is meaningless for master nodes.

Next, we discuss how to protect query privacy. Let 〈*T*, [*a, b*]〉 be a range query, where *T* is the time slot and [*a, b*] is the range of data in which the user is interested. Construct the range's *BFcode* of *m* bits by rules: hash each positive integer in [*a, b*] to *k* positions by hash functions *h*_1_, …,*h_k_*, and set these positions of the code to 1. In other words, the *BFcode* of the range is the union of the *BFcodes* of all positive integers in [*a, b*]. The message that the sink issues to the master node *M* is:

$$\text{sink}\to M:\langle T,BF_{\left[a,b\right]}\rangle$$

where *BF*_[_*_a,b_*_]_ represents the *BFcode* of the query range [*a, b*]. Without the right hash functions, the master node infers nothing about the query range.

### 5.3. Membership Test

*BFcodes* not only preserve the privacy of data and queries, but also enable master nodes to find required data without revealing any private information. Master nodes test the membership according to Theorem 1 (proven in Appendix A).

#### Theorem 1

*Given a range* [*a, b*] *and data d, BF_d_ and BF*_[_*_a, b_*_]_ *are respectively referred to the BFcode of d and the BFcode of* [*a, b*] *using k hash functions h*_1_,…, *h_k_. If BF_d_* ∩ *BF*_[_*_a, b_*_]_ ≠ *BF_d_, there must be d* ∉ [*a, b*].

However, the negative of Theorem 1 is not always true. That is, if *BF_d_* ∩ *BF_[a, b]_* ≠ *BF_d_*, there may be d ∉ [*a, b*], i.e., false positive. For instance, in Figure 2, the range is [11,15]. There is *BF*_16_ ∩ *BF*_[11,15]_ = *BF*_16_, but 16 ∉ [11,15]. As already mentioned in Section 4, a few false positives are allowable. The probability *p_p_* that data are considered to be in the query range after membership test is:

$$p_{p}=\prod_{i=1}^{k}\left(1-{\left(\prod_{j=1}^{k}\left(1-\frac{j}{m-i+1}\right)\right)}^{\left|b-a+1\right|}\right)$$

where *m* is the length of a *BFcode*. The derivation is detailed in Appendix B.

Receiving a query 〈*T*, *BF*_[_*_a,b_*_]_〉, master nodes begin to determine two bounds, denoted as *α* and *β*, for each sensor node *s_i_* with reference to Equations (1) and (2). Obviously, *d_α_* denotes the lower bound of data satisfying Equation (1), and *d_β_* denotes the upper bound of data satisfying Equation (2). It should be noticed that false positives may be generated during the membership test process, which is further improved in Section 7.

(1) $$\begin{array}{r} BF_{d_{\alpha }}\cap BF_{\left[a,b\right]}=BF_{d_{\alpha }}\\ \forall \lambda (\lambda <\alpha ),BF_{d_{\lambda }}\cap BF_{\left[a,b\right]}\neq BF_{d_{\lambda }} \end{array}$$

(2) $$\begin{array}{r} BF_{d_{\beta }}\cap BF_{\left[a,b\right]}=BF_{d_{\beta }}\\ \forall \lambda (\lambda >\beta ),BF_{d_{\lambda }}\cap BF_{\left[a,b\right]}\neq BF_{d_{\lambda }} \end{array}$$

The message that the master node *M* sends to the sink depends on 〈*α, β*〉 as follows:

- If *α* < *β*, that means the sensor node *s_i_* has multiple data in [*a, b*]. The message that *M* sends to the sink is:
  $$\begin{array}{c} M\to \text{sink}:i,\{E(d_{\alpha -1}\|\alpha -1),E(d_{\alpha }\|\alpha ),\ldots ,E(d_{\beta }\|\beta ),E(d_{\beta +1}\|\beta +1)\}\\ \left\{BF_{d_{\alpha -1}},BF_{d_{\alpha }},BF_{d_{\beta }},BF_{d_{\beta +1}}\right\} \end{array}$$
  where {*d_α_*, …, *d_β_*} is the result set, while {*d_α_*_–1_, *d_β_*_+1_} is the verification set.
- If *α* = *β*, that means the sensor node si has only one datum in [*a, b*]. The message that *M* sends to the sink is:
  $$\begin{array}{c} M\to \text{sink}:i,\{E(d_{\alpha -1}\|\alpha -1),E(d_{\alpha }\|\alpha ),E(d_{\alpha +1}\|\alpha +1)\}\\ \left\{BF_{d_{\alpha -1}},BF_{d_{\alpha }},BF_{d_{\alpha +1}}\right\} \end{array}$$
  where {*d_α_*} (*d_α_* = *d_β_*) is the result set, while {*d_α_*_–1_, *d_α_*_+1_} is the verification set.
- If *α* > *β* (actually *α* = *θ* + 1), that means all data of the sensor node *s_i_* are outside of [*a, b*]. The message that *M* sends to the sink is:
  $$M\to \text{sink}:i,\{E(d_{\alpha }\|\alpha ),E(d_{\beta }\|\beta )\},\{BF_{d_{\alpha }},BF_{d_{\beta }}\}$$
  where {*d_α_, d_β_*} is the verification set.

### 5.4. Integrity Verification

If each master node submits its local result honestly, the sink will obtain a correct final result. However, in a malicious environment, master nodes are attractive to adversaries. The compromised master node may submit the wrong local result to the sink. As a result, it is indispensable for the sink to verify the local results.

For the sensor node *s_i_*, let {*E*(*d_α_*_–1_‖*α* − 1),…, *E*(*d_β_*_+1_‖*β*+ 1)}{*BF_dα_*_–1_,…, *BF_dβ_*_+1_} be the message that the master node *M* sends to the sink. Thus, *RS* = {*d_α_*, …, *d_β_*} is the local result calculated based on the membership test, and *VS* = {*d_α_*_–1_, *d_β_*_+1_} is the verification set. Given a range query 〈T, *BF*_[_*_a,b_*_]_〉, result integrity is verified under various circumstances:

- If the encrypted part in the message cannot be decrypted using the right key, or the *BFcode* in the message is not equal to the correct one, the sink can know that the corresponding data are unauthentic; because only the authentic data are able to be decrypted by the right key and the *BFcode* should be equal to the correct one.
- If a datum *d* is in *RS* and its *BFcodes*, denoted as *BF_d_*, satisfy *BF_d_* ∩ *BF*_[_*_a,b_*_]_ ≠ *BF_d_*, the sink can detect this error. Because for any data *d* in *RS*, there must be *BF_d_* ∩ *BF*_[_*_a,b_*_]_ = *BF_d_*.
- If all orders of *d_α_*_–1_,*d_α_*,…,*d_β_,d_β_*_+1_ cannot keep continuity, the sink can discover this error. Because these orders increase continuously.
- If the master node returns nothing for the sensor node *s_i_*, the sink can find that this master node is compromised. Because the master node is required to submit {*E*(*d_α_*‖*α*), *E*(*d_β_*‖*β*)}, {*BF_dα_,BF_dβ_*}, even if there is no data in [*a, b*], besides, *α* = (*θ* + 1) and *β* = *θ*. If these two equalities do not hold simultaneously, the sink can know that the local result is incomplete.

Following the above rules, result integrity is verified and the compromised master node is detected.

### 5.5. Computational Complexity

We next present the computational complexity of PRQ. We report the time complexity of operations in each sensor node for data privacy preservation and the time complexity of operations in each master node for membership testing.

For any datum, the sensor node generates an encrypted part and a *BFcode*. It takes *O*(*k*) time to encode each datum to a *BFcode*, where *k* is the number of hash functions. Additionally, it generates an encrypted part with *O*(1) time by the encryption function. Assume the sensor node collects *θ* data. The overall time complexity of operations in each sensor node for data privacy preservation is *O*(*θ* · *k*).

For any encrypted data, the master node tests membership with *O*(*m*) time by comparing the *BFcode* of the data with the *BFcode* of the query, where *m* is the number of bits in a *BFcode*. Assume the master node receives *N BFcodes*. The overall time complexity of operations in each master node for membership testing is *O*(*N* · *m*).

## 6. Collusion-Aware Privacy-Preserving Range Query

Imagine a scenario where a sensor node colludes with its master node. The master node obtains hash functions stored in the sensor node and then uses these functions to infer the original data and queries from their corresponding *BFcodes* through enumeration methods. Therefore, collusion attacks between sensor nodes and master nodes will destroy network security and should be prevented effectively.

PRQ offers the preservation of privacy and integrity, but it is incapable of resisting collusion attacks. On the basis of PRQ, we propose a series of collusion-aware privacy-preserving range query protocols (CPRQ), which overcome the shortcoming of PRQ.

### 6.1. Confusion-Based Collusion-Aware Privacy-Preserving Range Query

In PRQ, the same hash functions *h*_1_,…,*h_k_* are shared with each sensor node and used to encode both data and queries. Once adversaries compromise any sensor node and the corresponding master node, private information will leak. Hence, different sensor nodes should use different hash functions to encode data, such that the impact of node collusion is limited.

Here, we design a confusion-based collusion-aware privacy-preserving range query protocol (c-CPRQ). c-CPRQ consists of the same steps as PRQ: system initialization, privacy preservation, membership testing and integrity verification. The differences between c-CPRQ and PRQ are in system initialization, privacy preservation and membership testing.

In system initialization, c-CPRQ defines the same data domain, hash function set *H* and key *k_i,t_* as PRQ. The query hash set (*QH*), a subset of *H*, is only secretly kept in the sink for encoding users' queries and |*QH*| = *k*. The node hash set (*NH_i_*) denotes the set of secret hash functions for encoding data of the sensor node *s_i_* and |*NH_i_*| = *k. NH_i_*, also a subset of *H*, is specified by the sink as follows: The sink selects *l* (0 < *l* < *k*) hash functions from *QH* and *k* − *l* hash functions from $\bar{QH}$. *NH_i_* is preloaded in *s_i_*. For example, assume *H* = {*h*_1_, *h*_2_,…, *h*_10_}, *k* = 4 and *l* = 3. First, the sink determines *QH* = {*h*_2_,*h*_5_,*h*_6_,*h*_9_}, and then, $\bar{QH}=\left\{h_{1},h_{3},h_{4},h_{7},h_{8},h_{10}\right\}$. The sink may select *NH*_1_ = {*h*_2_,*h*_5_,*h*_6_,*h*_1_} for the sensor node *s*_1_ and *NH*_2_ = {*h*_5_,*h*_6_,*h*_9_,*h*_3_} for the sensor node *s*_2_.

In privacy preservation, instead of using the identical hash functions for encoding, we use *NH_i_* and *QH* to construct the *BFcodes* of data of the sensor node *s_i_* and the *BFcodes* of queries, respectively. Different sensor nodes employ different hash functions, which results in two consequences: (1) the same data collected by different sensor nodes may be encoded to different *BFcodes;* (2) the same *BFcode* constructed by different sensor nodes may be derived from different data. Since the sensor node knows nothing except its own hash functions, its master node cannot infer the original data and the query range based on their *BFcodes*, even if it colludes with the sensor node. c-CPRQ protects privacy under node collusion conditions.

If c-CPRQ adopts the same membership test of PRQ, false negatives will be produced. That is because Theorem 1 used in the membership test of PRQ assumes that *NH_i_* = *QH* for each sensor node *s_i_*. In fact, *NH_i_* ≠ *QH* in c-CPRQ. To eliminate false negatives, we present a relaxed membership test according to Theorem 2 (proven in Appendix C).

#### Theorem 2

*Given a range* [*a, b*] *and data d, BF_d_ is referred to the BFcode of d using the hash function set NH, and BF*_[_*_a, b_*_]_ *is referred to the BFcode of* [*a, b*] *using the hash function set QH. Let η represent the number of 1-bits in BFd* ∩ *BF*_[_*_a, b_*_]_ *and IH* = *NH* ∩ *QH. If η* < |*IH*|, *there must be d* ∉ [*a, b*].

The probability *p_c_* that data are regarded as the members of the query range after membership testing is:

$$p_{c}=\overset{\left|NH\right|}{\underset{k=\left|IH\right|}{\text{∑}}}\left(\left(\begin{array}{c} \left|NH\right|\\ k \end{array}\right)*\prod_{i=1}^{k}\left(1-{\left(\prod_{j=1}^{\left|QH\right|}\left(1-\frac{j}{m-i+1}\right)\right)}^{\left|b-a+1\right|}\right)\right)$$

where *m* is the length of a *BFcode*. The derivation is similar to *p_p_*.

The master node processes the query 〈*T, BF*_[_*_a,b_*_]_〉 depending on Theorem 2. For each sensor node *s_i_*, the master node tries to find two bounds *α* and *β* according to Equations (3) and (4), where *η_i_* represents the number of 1-bits in *BF_di_* ∩ *BF*_[_*_a,b_*_]_ and *IH* = *NH_i_* ∩ *QH*. After determining *α* and *β*, the result that the master node sends to the sink is constructed following the same rules in PRQ.

(3) $$\begin{array}{r} \eta_{\alpha }\geq \left|IH\right|\\ \forall \lambda (\lambda <\alpha ),\eta_{\lambda }<\left|IH\right| \end{array}$$

(4) $$\begin{array}{r} \eta_{\beta }\geq \left|IH\right|\\ \forall \lambda (\lambda >\beta ),\eta_{\lambda }<\left|IH\right| \end{array}$$

The sink knows any *NH_i_*. After receiving the local results from master nodes, the sink starts to verify result integrity according to the integrity verification of PRQ.

False negatives are avoided in c-CPRQ based on Theorem 2. However, since *\NH_i_* ∩ *QH*| < |*NH_i_*|, more data not in [*a, b*] are transmitted by master nodes, thus causing the growth of false positives. This problem is settled in the next subsection.

### 6.2. l-Uncertainty Collusion-Aware Privacy-Preserving Range Query

To overcome the shortcoming of c-CPRQ, we put forward an *l*-uncertainty collusion-aware privacy-preserving range query protocol (u-CPRQ). The differences between u-CPRQ and c-CPRQ are in the system initialization and membership testing.

In system initialization, the sink chooses the query hash set (*QH*) from *H* and determines the node hash set (*NH_i_*) for each sensor node *s_i_* by randomly selecting at least l (0 < l ≤ |*QH*|) hash functions from *QH*, where *l* is a system parameter. *NH_i_* is preloaded in *s_i_*. |*NH_i_*| may be assigned to an arbitrary value in {*l, l* +1,.., |*QH*| − 1, |*QH*|}. Moreover, the hash functions in *NH_i_* may be an arbitrary combination of |*NH_i_*| hash functions in *QH*. Thus, |*NH_i_*| and *NH_i_* are both uncertain. For example, assume *H* = {*h*_1_, *h*_2_,…, *h*_10_}, *k* = 4 and *l* = 2. First, the sink determines |*QH*| = {*h*_2_,*h*_5_,*h*_6_,*h*_9_}. Then, the sink may select *NH*_1_ = {*h*_2_,*h*_5_} for the sensor node *s*_1_ and *NH_2_* = {*h*_5_,*h*_6_,*h*_9_} for the sensor node *s*_2_.

In privacy preservation, *NH_i_* is used to encode data of the sensor node *s_i_*, while *QH* is used to construct the *BFcodes* of queries.

Unlike c-CPRQ, u-CPRQ uses the same membership test of PRQ. Additionally, the probability that data belong to the query range after membership testing is:

$$p_{u}=\overset{\left|NH\right|}{\underset{i=1}{\text{∑}}}\left(1-{\left(\prod_{j=1}^{\left|QH\right|}\left(1-\frac{j}{m-i+1}\right)\right)}^{\left|b-a+1\right|}\right)$$

where *m* is the length of a *BFcode*. The derivation is similar to *p_p_*.

After finding two bounds 〈*α, β*〉, master nodes return local results to the sink. Finally, the sink checks result integrity. It is noted that |*NH_i_*| ≤ |*QH*|, so there is *p_u_* < *p_c_, i.e.*, false positives decrease.

### 6.3. Computational Complexity

We here discuss the computational complexity of c-CPRQ and u-CPRQ. We also report the time complexity of operations in each sensor node for data privacy preservation and the time complexity of operations in each master node for membership testing. Let *θ* denote the average number of data collected by the sensor node, *N* denote the average number of data received by the master node, *k* denote the number of hash functions and *m* denote the number of bits in a *BFcode*.

Similar to PRQ, in c-CPRQ, the sensor node needs to generate an encrypted parts and a *BFcodes* for each datum. The overall time complexity of operations in each sensor node for data privacy preservation is *O* (*θ* · *k*). The master node tests membership with *O* (*m*) time. The overall time complexity of operations in each master node for membership testing is *O*(*N* · *m*).

u-CPRQ is different from c-CPRQ in system initialization and membership testing. The sensor node encodes each datum with *O*(*l*) time instead of *O*(*k*) time, where *l*(0 < *l* ≤ *k*) is the system parameter and denotes the number of hash functions for data encoding. The overall time complexity of operations in each sensor node for data privacy preservation is *O*(*θ* ·*l*). The master node uses the membership test of PRQ. Therefore, the overall time complexity of operations in each master node for membership testing is *O*(*N* · *m*).

## 7. Schemes for Improvements

Given a certain query range, false positives decrease, but the cost of communication and storage grows as the size of a *BFcode* increases. In this section, two schemes are proposed to reduce communication cost and false positives. To simplify the description, we take PRQ as an example, but the series of CPRQ protocols can be improved by these schemes.

### 7.1. Data Compression for Lower Communication Cost

In PRQ, it is difficult to balance efficiency and accuracy. For one thing, fewer false positives require a larger size of a *BFcode* and higher communication cost for sensor nodes. For another, the size of messages sent by sensor nodes is expected to be as short as possible in order to lessen communication overhead and prolong the network lifetime.

It can be observed that the *BFcode* is filled with a few 1-bits and an enormous amount of 0-bits, so that data compression [34] is appropriate to find the tradeoff between efficiency and accuracy. Two efficient lossless compression schemes are provided as follows.

- Absolute 1-position compression: The *BFcode* can be represented by a set of positions of 1-bits, rather than the 0-1 string. For example, the *BFcode* 00001000000000011000000000000010 with four hash functions is represented by 〈5,16,17, 31〉, where 5, 16, 17 and 31 are the positions of 1-bits in the *BFcode*. Thus, instead of 32 bits, (⌊log_2_ 5⌋ + 1) + (⌊log_2_ 16⌋ + 1) + (⌊log_2_ 17⌋ + 1) + (〈log_2_ 31〉 +1) = 18 bits are enough to represent the original *BFcode*, and the compression ratio is 32/18 ≈ 1.78. Equations (5) and (6) respectively show the maximum and minimum number of bits required by absolute 1-position compression, where *k* denotes the number of hash functions, *P^j^* denotes the position of the *j*-th 1-bit in the *BFcode* and *m* denotes the length of an uncompressed *BFcode. B_max_* bits are needed when all *k* 1-bits fall into the last *k* positions of the *BFcode*, and *B_min_* bits are needed when all *k* positions are hashed to the first *k* positions of the *BFcode*.
  (5) $$B_{\text{max}}=\max\left\{\overset{k}{\underset{j=1}{\text{∑}}}\left(\left\lfloor \log_{2}P^{j}\right\rfloor +1\right)\right\}=\overset{m}{\underset{i=m-k+1}{\text{∑}}}\left(\left\lfloor \log_{2}i\right\rfloor +1\right)$$
  (6) $$B_{\text{min}}=\min\left\{\overset{k}{\underset{j=1}{\text{∑}}}\left(\left\lfloor \log_{2}P^{j}\right\rfloor +1\right)\right\}=\overset{k}{\underset{i=1}{\text{∑}}}\left(\left\lfloor \log_{2}i\right\rfloor +1\right)$$
- Differential 1-position compression: The *BFcode* can be represented by differences between two adjacent positions of 1-bits. For instance, the same *BFcode* mentioned in absolute 1-position compression is encoded to 〈5,11,1,14〉, where 5 is the position of the first 1-bit, while 11, 1 and 14 are the position differences between the second 1-bit and the first 1-bit, the third 1-bit and the second 1-bit and the forth 1-bit and the third 1-bit, respectively. It needs (⌊log_2_ 5⌋ + 1) + (⌊log_2_ 11⌋ + 1) + (⌊log_2_ 1⌋ + 1) + (⌊log_2_ 14⌋ + 1) = 12 bits, and the compression ratio 32/12 ≈ 2.67, which is larger than that of absolute 1-position compression. The maximum number of bits required by differential 1-position compression is illustrated in Equation (7), where *P*^0^ = 0. $B_{\max}^{\prime }$ bits are needed when all *k* 1-bits are distributed uniformly in the *BFcode*. The minimum number of bits is the same as that in absolute 1-position compression.

(7) $$B_{\text{max}}^{\prime }=\max\left\{\overset{k}{\underset{j=1}{\text{∑}}}\left(\left\lfloor \log_{2}\left(P^{j}-P^{j-1}\right)\right\rfloor +1\right)\right\}=k\times \left(\left\lfloor \log_{2}\left(\frac{m}{k}\right)\right\rfloor +1\right)$$

The two compression algorithms compress a *BFcode* with *O*(*m*) time by scanning an uncompressed *BFcode* of *m* bits. Each sensor node compresses *θ* data with *O*(*θ* · *m*) time. After data compression, the size of messages is shortened and communication cost is cut down dramatically for sensor nodes. Besides, these two compression schemes are lossless. Thus, master nodes can decompress accurate *BFcodes*.

### 7.2. Multiple BFcodes for Fewer False Positives

Assume the query is denoted by 〈*T*, [*a, b*]〉. In PRQ, [*a, b*] is converted to a single *BFcode BF*_[_*_a,b_*_]_, which is the union of the *BFcodes* of data in [*a, b*]. If [*a, b*] contains more data, more positions of *BF*_[_*_a,b_*_]_ will be set to 1. The positions, to which data not in [*a, b*] are hashed, are likely to be set to 1 by the *BFcodes* of data in [*a, b*]. As a result, this leads to undesirable false positives.

To reduce false positives, PRQ is improved by constructing multiple *BFcodes* for the query range [*a, b*]. The basic idea is: divide all data in [*a, b*] into *w* disjoint groups *G*_1_,…, *G_w_* (*G_i_* ∩ *G_j_* = Ø, *i* ≠ *j*) and then construct a *BFcode*, denoted as $BF_{\left[a,b\right]}^{i}$, for each group *G_i_*. Therefore, [*a, b*] is transformed to *w BFcodes*. If the original *BFcode* is of *m* bits, using *w BFcodes* to represent [*a, b*] is equivalent to assigning *w × m* bits for the original single *BFcode*. The message that the sink sends to the master node *M* is changed to:

$$\text{sink}\to M:T,\left\{BF_{\left[a,b\right]}^{1},BF_{\left[a,b\right]}^{2},\ldots ,BF_{\left[a,b\right]}^{w}\right\}$$

The data of sensor nodes are still encoded to a *BFcode* in the way mentioned in Section 5. When a query $\langle T,\left\{BF_{\left[a,b\right]}^{1},BF_{\left[a,b\right]}^{2},\ldots ,BF_{\left[a,b\right]}^{w}\right\}\rangle$ arrives, master nodes begin to search for the bounds *α* and *β* according to Equations (8) and (9).

(8) $$\begin{array}{r} \exists k(1\leq k\leq w),BF_{d_{\alpha }}\cap BF_{\left[a,b\right]}^{k}=BF_{d_{\alpha }}\\ \forall \lambda (\lambda <\alpha ),\forall_{j}(1\leq j\leq w),BF_{d_{\lambda }}\cap BF_{\left[a,b\right]}^{j}\neq BF_{d_{\lambda }} \end{array}$$

(9) $$\begin{array}{r} \exists k(1\leq k\leq w),BF_{d_{\beta }}\cap BF_{\left[a,b\right]}^{k}=BF_{d_{\beta }}\\ \forall \lambda (\lambda <\beta ),\forall j(1\leq j\leq w),BF_{d_{\lambda }}\cap BF_{\left[a,b\right]}^{j}\neq BF_{d_{\lambda }} \end{array}$$

Assume *m* is the number of bits in a *BFcode*. Using the multiple *BFcodes* scheme, each master node needs *O*(*m* · *w*) time to test the membership of each datum, while false positives are reduced.

The partition of *w* groups affects the performance of eliminating false positive. Here, we give two schemes. The first scheme separates [*a, b*] uniformly into *w* consecutive sub-ranges, which is called consecutive multiple *BFcodes* (CMB). The second one inserts data into a certain group with a probability 1/*w*, which is called random multiple *BFcodes* (RMB). The two schemes are evaluated in Section 9.

## 8. Theoretical Analysis

Section 3 describes the requirements for a good privacy-preserving range query protocol. Result accuracy is affected by the probability of false positives, which is discussed in Sections 5 and 6. In this section, we analyze efficiency and privacy.

### 8.1. Efficiency Analysis

In WSNs, communication cost is the main factor to influence the efficiency of a protocol. As in [2–9], we analyze the proposed protocol in terms of communication cost, which is defined as the communication consumption in bits during query processing. It is generated during data submission from sensor nodes to master nodes and result submission from master nodes to the sink, *i.e.*, communication cost of sensor nodes and communication cost of master nodes. The lower the communication cost, the higher the efficiency.

Assume *N* denotes the number of sensor nodes, *l_e_* denotes the average length of an encrypted part, *m* denotes the size of a *BFcode, θ* denotes the average number of data collected by a sensor node and *p* denotes the probability that data are considered to be in the query range.

#### 8.1.1. Communication Cost of Sensor Nodes

In any protocol, sensor nodes transmit encrypted data and associated *BFcodes* to the nearest master nodes, so the communication cost of sensor nodes is:

(10) $$N*\left(l_{e}+m\right)*(\theta +2)$$

#### 8.1.2. Communication Cost of Master Nodes

The rules of membership testing influence the accuracy of 〈*α, β*〉, and thus, the accuracy of 〈*α, β*〉 affects the communication cost of master nodes. Three cases should be considered: (1) if *α* > *β*, the expectation of communication cost is *N* × 2 × (*l_e_* + *m*) × (1 − *p*)*^θ^*; (2) if *α* = *β*, the expectation of communication cost is *N* × 2 × (*l_e_* + *m*) × (1 − *p*)*^θ^*^−1^ × *p*; and (3) if *α* < *β*, the expectation of communication cost is $N\times \left(l_{e}+m\right)\times {\text{∑}}_{i=1}^{\theta }\left({\left(1-p\right)}^{i-1}\times p^{2}\times {\text{∑}}_{j=i+1}^{\theta }\left({\left(1-p\right)}^{\theta -j}\times \left(j-i+3\right)\right)\right)$. Therefore, the total communication cost of master nodes is:

(11) $$N\times \left(l_{e}+m\right)\times \left(2{\left(1-p\right)}^{\theta -1}+\overset{\theta }{\underset{i=1}{\text{∑}}}\left({\left(1-p\right)}^{i-1}\times p^{2}\times \overset{\theta }{\underset{j=i+1}{\text{∑}}}\left({\left(1-p\right)}^{\theta -j}\times \left(j-i+3\right)\right)\right)\right)$$

Since the accuracies of 〈*α, β*〉 are different in different protocols, *p* is protocol-specific.

### 8.2. Privacy Analysis

First, we analyze privacy under non-collusion conditions and then discuss privacy under node collusion conditions later.

#### 8.2.1. Privacy under Non-Collusion Conditions

- Data privacy: In any protocol, original data are stored in the encrypted format and will be disclosed if master nodes have the right key. If the master node knows the length *l_k_* of each key, the probability that it guesses the correct data is:
  (12) $$2^{-l_{k}}$$
  If *l_k_* is large enough, the probability is negligible. Furthermore, keys change constantly at different epochs, such that past keys are invalid at the current epoch.
  There is another way to obtain original data. If the master node is aware of the hash functions *NH* for data *d* and the associated *BF_d_*, it can derive $\left(\begin{array}{c} m^{\prime }\\ \left|NH\right| \end{array}\right)\text{BFcodes}$ from *BF_d_*, where *m′* is the number of 1-bits in *BF_d_*. Each *BFcode* may be mapped from |*NH*|! data. Therefore, the probability that the master node guesses the correct data is:
  (13) $${\left(\left(\begin{array}{c} m^{\prime }\\ \left|NH\right| \end{array}\right)\times \left|NH\right|!\right)}^{-1}$$
- Query privacy: In any protocol, given a query 〈*T*, [*a, b*]〉, the sink transforms [*a, b*] to *BF*_[_*_a,b_*_]_. Because *BF*_[_*_a,b_*_]_ is the union of many *BFcodes* and each *BFcode* cannot be distinguished from others, *BF*_[_*_a,b_*_]_ is meaningless for the master node. Let *QH* be the hash functions for [*a, b*]. If the master node knows *QH*, it can derive $\left(\begin{array}{c} m^{\prime }\\ \left|QH\right| \end{array}\right)\text{BFcodes}$ from *BF*_[_*_a,b_*_]_, where *m′* is the number of 1-bits in *BF_a,b_*_]_. Each *BFcode* may be hashed from |*QH*|! data. Thus, the probability that the master node guesses the correct query range is:
  (14) $${\left(\begin{array}{c} \left(\begin{array}{c} m^{\prime }\\ \left|QH\right| \end{array}\right)\times \left|QH\right|!\\ 2 \end{array}\right)}^{-1}$$
  If *QH* is unavailable, the probability can be neglected.

#### 8.2.2. Privacy under Collusion Conditions

If master nodes collude with sensor nodes, they can obtain the hash functions and deduce original data and the query range by enumeration methods. The key point is to keep adversaries from the hash functions. As mentioned in Section 6, PRQ cannot resist node collusion. We analyze the series of CPRQ protocols.

Assume *H* is the hash function pool; *NH* and *QH* are the hash functions for data and queries, respectively. Since different sensor nodes have different *NH*, each sensor node cannot infer the real data of others according to the *BFcodes*. However, *H* can be deduced by compromising enough sensor nodes; thus, *QH* or *NH* may be further guessed by choosing |*QH*| or |*NH*| hash functions from *H*. On the premise of knowing *H*, the probability that *QH* is deduced and the probability that *NH* is deduced are ${\left(\begin{array}{c} \left|H\right|\\ \left|QH\right| \end{array}\right)}^{-1}$ and ${\left(\begin{array}{c} \left|H\right|\\ \left|NH\right| \end{array}\right)}^{-1}$, respectively. Now, we discuss how many sensor nodes are needed to figure out *H*. This is an instance of the coupon collector's problem [35].

Let *x* be the number of collusive sensor nodes and $N_{H}^{x}$ be the number of different hash functions obtained by these *x* sensor nodes. Assume $N_{H}^{x}-N_{H}^{x-1}=1$, i.e., $N_{H}^{1}=\left|NH\right|$, $N_{H}^{2}=\left|NH\right|+1,\ldots ,$ $N_{H}^{x}=\left|NH\right|+x-1$. Equation (15) indicates the probability that *x* nodes collude to gain |*NH*| + *x* − 1 different hash functions after *x* − 1 nodes get |*NH*| + *x* − 2 different ones.

(15) $$p_{x}^{b}=\{\begin{array}{ll} 1 & ifx=1\\ \left(\begin{array}{c} \left|NH\right|\\ 1 \end{array}\right)\times \frac{\left(\left|H\right|-\left(\left|NH\right|+x-2\right)\right)\times \prod_{v=0}^{\left|NH\right|-2}\left(\left|NH\right|+x-2-v\right)}{\prod_{u=0}^{\left|NH\right|-1}\left(\left|H\right|-u\right)} & if1<x\leq \left|H\right|-\left|NH\right|+1, \end{array}$$

where |*NH*| is protocol specific.

To get *H*, the expected number of collusive sensor nodes, denoted as 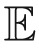
(*X*), is:

(16) $$E\left(X\right)=\overset{\left|H\right|-\left|NH\right|+1}{\underset{x=1}{\text{∑}}}\frac{1}{p_{x}^{b}}.$$

Since limited sensor nodes and master nodes could be compromised by adversaries, if 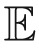
(*X*) is too large, collusion attacks will fail.

## 9. Evaluation

As mentioned in Section 2, node collusion is not taken into account in previous related work. Therefore, we first analyze the parameters of improvements and then evaluate PRQ by comparing with Encoding [2], ST-crosscheck [3] and SafeQ [6] under non-collusion conditions and, finally, contrast the series of CPRQ protocols with PRQ considering collusion attacks. Performance is thoroughly evaluated on the same two-tiered model in terms of efficiency, accuracy and privacy.

All protocols are implemented on OMNet++4.1, a widely-used simulator for WSNs. The network is set to 400 m × 400 m. Sensor nodes are uniformly deployed in the network, and the number of sensor nodes changes from 200 to 600. Assume that the network is separated into four identical cells and a master node is at the center of each cell. The transmission radius of each sensor node is 50 m. The real dataset, LUCE (Lausanne Urban Canopy Experiment) [36], is used in the experiments. LUCE is a measurement campaign, which took place on the EPFLcampus from July 2006, to May 2007, and aimed at better understanding micrometeorology and atmospheric transport in the urban environment. It contains 97 sensor nodes, but we need more sensor nodes. Therefore, we use data collected in LUCE. We adopt the revised Bernstein hash [37], which holds the property that for any hash function *h_i_* and *h_j_* (*i* ≠ *j*), there is *h_i_*(*d*) = *h_j_*(*d*). Unless there is a particular specification, our protocols encode data and queries to a *BFcode* of 128 bits by using four hash functions, while the data domain is partitioned into 10 buckets for Encoding and ST-crosscheck.

### 9.1. Parameters of Improvements

To simplify the description, we also take PRQ as an example, but these schemes are able to improve the series of CPRQ protocols.

#### 9.1.1. Data Compression

**Figure 3.**
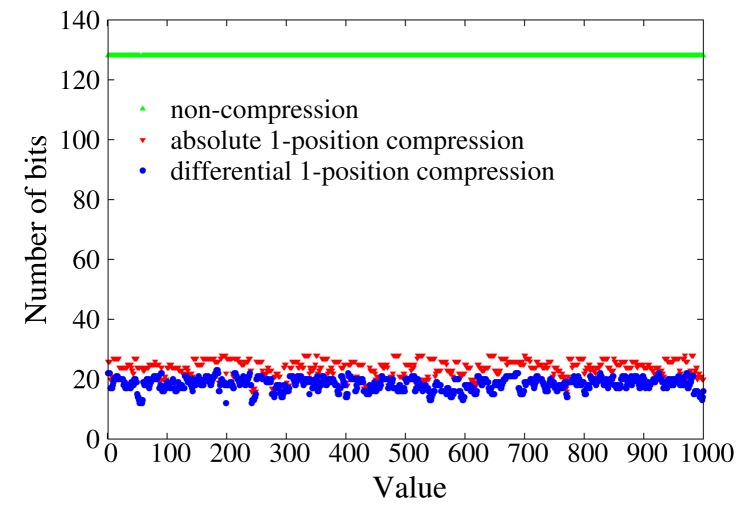
Comparison of data compression.

Data compression is theoretically confirmed to be efficient enough to shorten the size of messages sent by sensor nodes in Section 7. The two proposed data compression schemes and the non-compression scheme are compared in Figure 3 by compressing *BFcodes* of integers in [0, 1000]. The size of an uncompressed *BFcode* is 128 bits, and 4 revised Bernstein hash functions [37] are used to encode data. Absolute 1-position compression compresses a *BFcode* to about 18 bits, while differential 1-position compression compresses that to about 24 bits. We define the compression ratio as the ratio of the number of bits of an uncompressed *BFcode* to the number of bits of a compressed *BFcode*. The larger the compression ratio, the shorter the message and the less the communication cost. The compression ratio of absolute 1-position compression and differential 1-position compression are about 7 and 5, respectively. It is obvious that differential 1-position compression can make messages shorter and save more on the communication cost. Our protocols adopt differential 1-position compression.

#### 9.1.2. Multiple *BFcodes*

Given a query range [*a, b*], result accuracy is affected by false positives. The probability of false positives is defined as the ratio of the number of unsatisfactory data in the result to the number of data in the result. The fewer the false positives, the better the accuracy.

**Figure 4.**
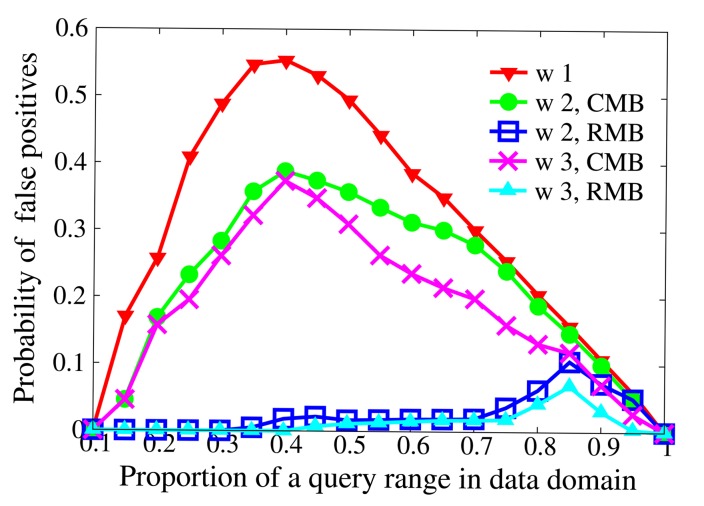
Impact of multiple *BFcodes* on false positives.

Figure 4 demonstrates the impact of multiple *BFcodes* on false positives. In this experiment, the size of a *BFcode* is 128 bits. The data domain is [0, 1000]. We randomly choose 10 ranges in the data domain for each proportion of a query range and take the average probability of false positives. *w* (*w* = 1,2, 3) denotes the number of *BFcodes* for queries. Consecutive multiple *BFcodes* (CMB) and random multiple *BFcodes* (RMB) are the proposed partition schemes of a query range.

Given *w*, the probability of false positives first increases and then declines. Sensor nodes generate the same data for different proportions of the query range [*a, b*]. When the proportion is small, few data are included in [*a, b*], such that few bits of *BF*_[_*_a,b_*_]_ are set to 1 and lots of unsatisfactory data are filtered out correctly. When the proportion increases to a certain extent, the probability of false positives peaks. The reason is that more bits of *BF*_[_*_a,b_*_]_ are set to 1, failing to filter unsatisfactory data out. As the proportion continues growing, more bits of *BF*_[_*_a,b_*_]_ are set to 1, but the probability of false positives decreases, because more and more data really belong to [*a, b*].

Given the partition scheme, the probability of false positives with *w* = 2, 3 is smaller than that with *w* = 1, because the scheme using *w BFcodes* potentially assigns *w*×*m* bits for the *BFcode* of [*a, b*]. For a certain *w*, RMB produces fewer false positives than CMB. This may be explained by the reason that adjacent data are likely to be hashed to similar positions, and RMB divides [*a, b*] randomly to eliminate the position correlation. It is found that the probability of false positives with *w* = 2 is similar to that with *w* = 3. In the meanwhile, the computational cost of master nodes increases as *w* grows. After comprehensive consideration, our protocols use the scheme with RMB and *w* = 2.

### 9.2. Performance Evaluation under Non-Collusion Conditions

#### 9.2.1. Efficiency

**Figure 5.**
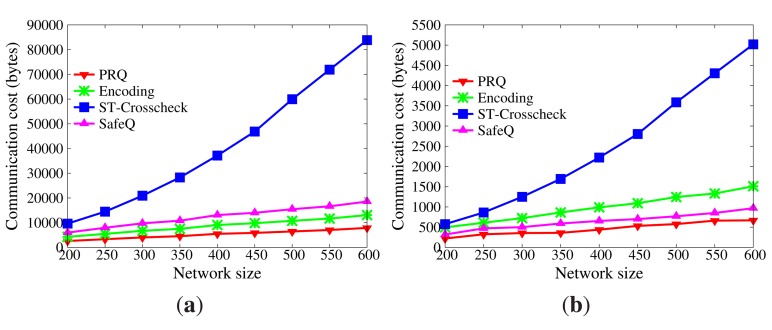
Impact of network size on communication cost without collusion attacks. (**a**) Sensor nodes; (**b**) master nodes.

In WSNs, communication is the dominant factor of energy consumption. The lower the communication cost, the better the efficiency Figure 5 displays the impact of network size on communication cost without collusion attacks (the submission period is 30 s). As the size of the network grows, more data are transmitted from sensor nodes to master nodes, and more data stored in master nodes satisfy the query range. Therefore, communication cost increases. Besides, the communication cost of sensor nodes goes far beyond that of master nodes, because master nodes only submit satisfactory data. It is evident that the communication cost of sensor nodes in PRQ is much less than that of other protocols. In ST-crosscheck, each bucket of sensor nodes attaches the bucket information of all neighbors, and its communication cost of sensor nodes is related to the product of the number of their neighbors and the square of the bucket number. In Encoding, the more the empty buckets, the more the attached codes. SafeQ constructs a prefix family for data as the attached code. Given the data *d*, their prefix family needs (⌊log_2_ *d*⌋ + 1)^2^ bits. However, PRQ just appends a compressed *BFcode* to the data.

**Figure 6.**
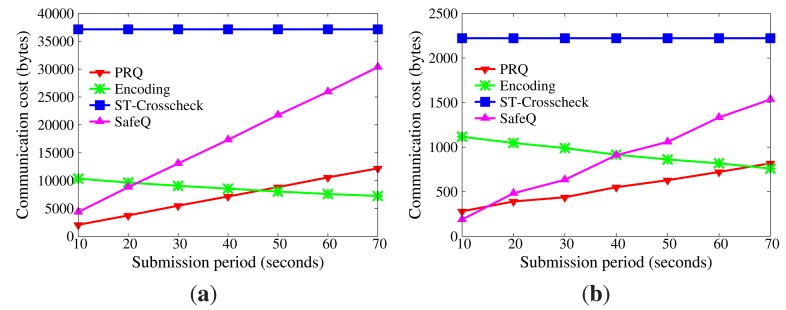
Impact of submission period on communication cost without collusion attacks. (**a**) Sensor nodes; (**b**) master nodes.

Figure 6 demonstrates the impact of submission period on communication cost without node collusion (the network size is 400). The submission period is defined as the interval time between two successive submissions. It is observed that the submission period has no effect on the communication cost of the sensor node in ST-crosscheck, because it is only related to the number of neighbors and the number of buckets. Due to the reduction of empty buckets, the communication cost of sensor nodes in Encoding decreases as the submission period increases, which is opposite to what occurs in PRQ and SafeQ. When the submission period is below 40 s, the communication cost of sensor nodes in PRQ is less than the others. When the submission period continues growing, the communication cost of sensor nodes in PRQ increases slowly. Each datum *d* in SafeQ needs to attach (⌊log_2_ *d*⌋ + 1) prefix-membership codes, whereas only one compressed *BFcode* is appended in our PRQ.

Under non-collusion conditions, PRQ saves more communication cost and energy consumption than others.

#### 9.2.2. Accuracy

Incomplete results can be detected in PRQ, Encoding, ST-crosscheck and SafeQ, but false positives are difficult to eliminate. The probability of false positives is defined the same as in Section 9.1.2. The lower probability of false positives, the higher the accuracy.

**Figure 7.**
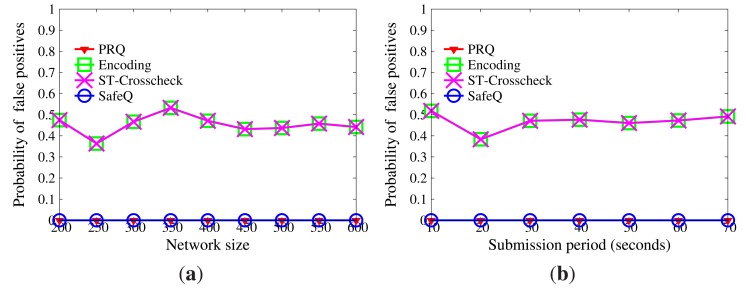
False positives without collusion attacks. (a) Impact of network size; (b) impact of submission period.

Figure 7 reveals how the probability of false positives is influenced by network size and submission period. The result of SafeQ is accurate, and there are no false positives. Encoding and ST-crosscheck are built on the bucketing technique, which has a great effect on accuracy. The more the buckets, the fewer the false positives, but the weaker the privacy. On account of multiple *BFcodes*, PRQ achieves accurate results, as SafeQ does. Besides, the network size and submission period have no impact on the probability of false positives.

#### 9.2.3. Privacy

**Figure 8.**
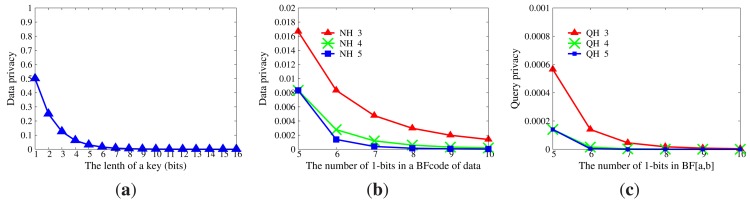
Privacy without collusion attacks. (**a**) Impact of key; (**b**) Impact of *NH;* (**c**) Impact of *QH*.

Data privacy will be disclosed if master nodes acquire either the correct key or the correct hash functions *NH* for data encoding, as mentioned in Section 8.2.1. The privacy of data is shown in Figure 8a,b. The probability that master nodes can decrypt data correctly is extremely tiny if the length of a key is large than 8 bits. The probability that master nodes can guess actual data upon their *BFcodes* and right hash functions *NH* is less than 0.01 when |*NH*| ≥ 4. Figure 8c displays the privacy intensity of queries. For a certain range [*a, b*], it is obvious that the probability that [*a, b*] is deduced from the *BF*_[_*_a,b_*_]_ can be neglected, even though the set of hash functions *QH* for query encoding is reachable.

### 9.3. Performance Evaluation under Collusion Conditions

In the following experiments, in PRQ, c-CPRQ and u-CPRQ, the number *k* of hash functions for query encoding is set to 4. Since the parameter *l* in c-CPRQ satisfies 0 < *l* < *k* and *l* in u-CPRQ satisfies 0 < *l* ≤ *k*, we set *l* = 3.

#### 9.3.1. Efficiency

Node collusion may be destructive. The series of CPRQ protocols not only preserves privacy and integrity, but also resists collusion attacks, while PRQ is disabled under collusion conditions.

**Figure 9.**
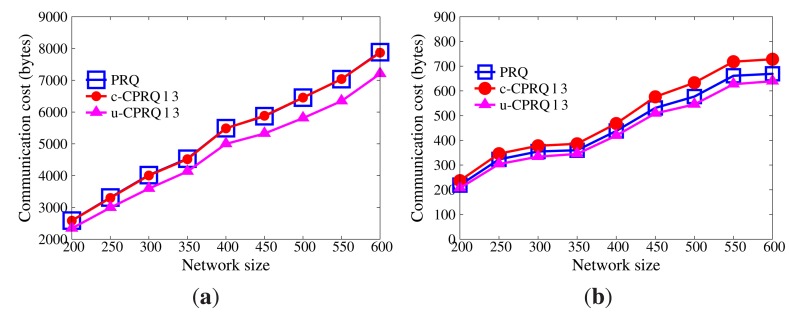
Impact of network size on communication cost with collusion attacks. (**a**) Sensor nodes; (**b**) master nodes.

**Figure 10.**
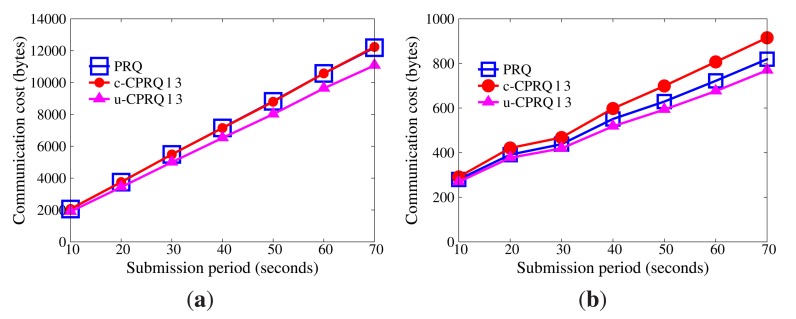
Impact of submission period on communication cost with collusion attacks. (**a**) Sensor nodes; (**b**) master nodes.

Figures 9 and 10 demonstrate the communication cost of the series of CPRQ protocols compared with PRQ. It is observed that the communication cost of sensor nodes in u-CPRQ is less than those in c-CPRQ and PRQ. Sensor nodes transmit all encrypted data and corresponding *BFcodes* to master nodes. Since the size of the encrypted part for each protocol is identical, the size of a compressed *BFcode* determines the communication cost of sensor nodes. Because the number of hash functions for data in u-CPRQ is less than those in c-CPRQ and PRQ, the size of a compressed *BFcode* in u-CPRQ is smaller than those of others. Besides, this shows that the communication cost of master nodes in PRQ is less than that in c-CPRQ, but is greater than that in u-CPRQ. Since c-CPRQ adopts a relaxed membership test, it produces more false positives than PRQ. The size of a compressed *BFcode* in u-CPRQ is smaller than that of PRQ, such that the communication cost of master nodes in u-CPRQ is less than that in PRQ.

#### 9.3.2. Accuracy

**Figure 11.**
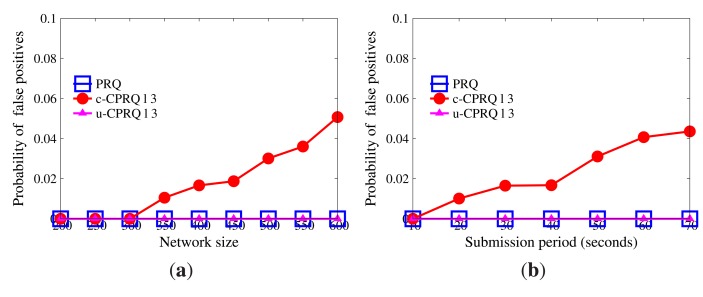
False positives with collusion attacks. (**a**) Impact of network size; (**b**) impact of submission period.

Given a range query, the definition of the probability of false positives is the same as that in Section 9.1.2. Figure 11 indicates the probability of false positives. It can be seen that the probability of false positives in both u-CPRQ and PRQ is 0%, while that in c-CPRQ increases with the growth of the network size and submission period. The reason is that the relaxed membership test in c-CPRQ brings in false positives, and there are no false positives generated in PRQ and u-CPRQ based on the precise bounds. However, u-CPRQ and c-CPRQ can resist collusion attacks, while PRQ cannot. c-CPRQ is also acceptable for users if the probability of false positives in c-CPRQ is small enough.

#### 9.3.3. Privacy

**Figure 12.**
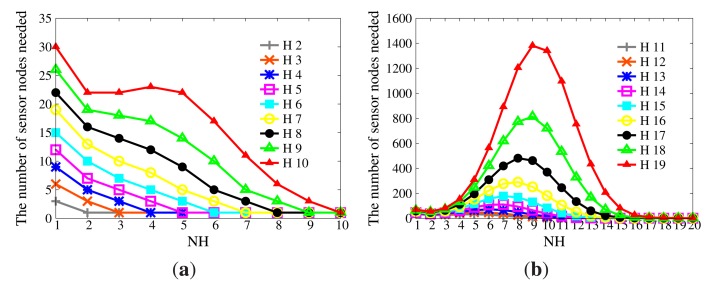
Expectation of the number of sensor nodes needed for a successful collusion. (**a**) |*H*| ∈ {2, 3, 4, 5, 6, 7, 8, 9, 10}; (**b**)|*H*| ∈ {11, 12, 13, 14, 15, 16, 17, 18, 19, 20}.

If there are sufficient collusive sensor nodes, *H* will be inferred based on *NH*. The expectation of the number of sensor nodes needed to infer *H* is provided in Figure 12. When |*H*| is large enough, it becomes a bell curve, whose axis of symmetry is the line |*NH*| = |*H*|/2. Thus, |*NH*| should be selected from the left side of the axis of symmetry in order to control the size of the compressed *BFcode*. To guarantee the availability of WSNs, the number of collusive sensor nodes should be limited. For example, the size of the network is 1000, and at most, 10% of sensor nodes will be compromised. Given |*H*| = 20, if |*NH*| = 4, at least 179 sensor nodes are needed, while if |*NH*| = 10, at least 2397 sensor nodes are needed. Due to the limited attack ability, it is impossible for adversaries to compromise so many sensor nodes. Therefore, the series of CPRQ can prevent collusion attacks.

From the above, c-CPRQ and u-CPRQ are robust to collusion attacks, while preserving privacy and integrity. Especially u-CPRQ outperforms PRQ in terms of efficiency, accuracy and privacy. If the probability of false positives is very small, c-CPRQ is also a good choice for users.

## 10. Conclusions

Privacy issues restrict the widespread adoption of WSNs and even threaten the security of the IoT. In this paper, we propose a privacy-preserving range query protocol, PRQ, and then present a series of collusion-aware privacy-preserving range query protocols: c-CPRQ and u-CPRQ. To the best of our knowledge, this paper is the first to take collusion attacks into account for a privacy-preserving range query in tiered WSNs. In our protocols, data and queries are represented by *BFcodes* to hide the original information, while result integrity is verified through ordinal relation among data. In the series of CPRQ protocols, sensor nodes use diverse hash functions to prevent node collusion. The performance of our proposals is evaluated by comparing with Encoding, ST-crosscheck and SafeQ. Theoretical analysis and simulation results demonstrate the high performance of our protocols in terms of efficiency, accuracy and privacy. In the future, we will focus on the privacy preservation of more complex queries, such as top-*k* and *k*NN, in two-tiered WSNs. For these two queries, the final result relies on global comparison information. It is challenging to compare data without information leakage and to achieve accurate results.

## Figures and Tables

**Table 1.** Notation.

**Symbol**	**Meaning**
*H*	hash function pool
*h_i_*	a hash function in *H*
*t*	an epoch
*T*	a time slot
*M*	a master node
*s_i_*	a sensor node with the unique identifier *i*
*k_i,t_*	a secret key of *s_i_* at epoch *t*
*d_j_*	the *j*-th data of *s_i_*
*BF_d_*	*BFcode* of data *d*
[*a, b*]	a query range
*BF*_[_*_a,b_*_]_	*BFcode* of range [*a, b*]
*E*()	an encryption function
*QH*	hash function set for queries
*NH_i_*	hash function set for data of *s_i_*

## Appendix

### A. Proof of Theorem 1

#### Proof

We prove the theorem using reduction to absurdity. Assume ∃*d′* ϵ [*a, b*] satisfying *BF_d′_* ∩ *BF*_[_*_a,b_*_]_ ≠ *BF_d′_*. Because *BF*_[_*_a,b_*_]_ = ∪*_d_*_ϵ|_*_a,b_*_|_*BF_d_*, we have:

$$\begin{array}{l} BF_{d^{\prime }}\cap BF_{\left[a,b\right]}\\ =BF_{d^{\prime }}\cap \left(\cup_{d\in \left[a,b\right]}BF_{d}\right)\\ =\cup_{d\in \left[a,b\right]}\left(BF_{d^{\prime }}\right)\cap BF_{d}\\ =\left(BF_{d^{\prime }}\cap BF_{d^{\prime }}\right)\cup \left(\cup_{d\in \left[a,b\right]\wedge d\neq d^{\prime }}\left(BF_{d^{\prime }}\right)\cap BF_{d}\right)\\ =BF_{d^{\prime }}\cup \left(\cup_{d\in \left[a,b\right]\wedge d\neq d^{\prime }}\left(BF_{d^{\prime }}\cap BF_{d}\right)\right)\\ =BF_{d^{\prime }} \end{array}$$

which contradicts the assumption. Thus, *d′* ∉ [*a, b*]. Therefore, if *BF_d_* ∩ *BF*_|_*_a,b_*_|_ ≠ *BF_d_*, there must be *d* ∉ [*a, b*].

### B. Derivation of p_p_

Given a large enough *m*, we can construct *k* hash functions *h*_1_, …, *h_k_*, holding the property that ∀*d, h_i_*(*d*) ≠ *h_j_*(*d*) (1 ≤ *i* ≠ *j* ≤ *k*), which is different from hash functions in [33].

The probability that the *i*-th 1-bit of *BF_d_* is 0 in *BF*_[_*_a,b_*_]_ is ${\left(\prod_{j=1}^{k}\left(1-\frac{j}{m-i+1}\right)\right)}^{\left|b-a+1\right|}$. In other words, the probability that the *i*-th 1-bit of *BF_d_* is 1 in *BF*_|_*_a,b_*_|_ is $1-{\left(\prod_{j=1}^{k}\left(1-\frac{j}{m-i+1}\right)\right)}^{\left|b-a+1\right|}$. The probability that all 1-bits of *BF_d_* are 1 in *BF*_[_*_a,b_*_]_ is $\prod_{i=1}^{k}\left(1-{\left(\prod_{j=1}^{k}\left(1-\frac{j}{m-i+1}\right)\right)}^{\left|b-a+1\right|}\right)$.

### C. Proof of Theorem 2

#### Proof

We prove the theorem using reduction to absurdity.

Assume ∃*d′* ∈ [*a, b*] satisfying *η* < |*I H*|. According to the construction of *BF*_[_*_a,b_*_]_, *BF*_[_*_a,b_*_]_ = ∪*_d_*_ϵ|_*_a,b_*_|_*BF_d_*. That means the positions to which *d′* is mapped are set to 1 in *BF*_|_*_a,b_*_|_. Let $BF_{X}^{H\#}$ denote the *BFcode* of *X* using hash function set *H*^♯^. Then, we have:

$$\begin{array}{l} BF_{d^{\prime }}\cap BF_{\left[a,b\right]}\\ =\left(BF_{d^{\prime }}^{IH}\cup BF_{d^{\prime }}^{NH\backslash IH}\right)\cap \left(BF_{\left[a,b\right]}^{IH}\cup BF_{\left[a,b\right]}^{QH\backslash IH}\right)\\ =\left(BF_{d^{\prime }}^{IH}\cap BF_{\left[a,b\right]}^{IH}\right)\cup \left(BF_{d^{\prime }}^{NH\backslash IH}\cap BF_{\left[a,b\right]}^{IH}\right)\\ \cup \left(BF_{d^{\prime }}^{IH}\cap BF_{\left[a,b\right]}^{QH\backslash IH}\right)\cup \left(BF_{d^{\prime }}^{NH\backslash IH}\cap BF_{\left[a,b\right]}^{QH\backslash IH}\right)\\ =BF_{d^{\prime }}^{IH}\cup \left(BF_{d^{\prime }}^{NH\backslash IH}\cap BF_{\left[a,b\right]}^{IH}\right)\\ \cup \left(BF_{d^{\prime }}^{IH}\cap BF_{\left[a,b\right]}^{QH\backslash IH}\right)\cup \left(BF_{d^{\prime }}^{NH\backslash IH}\cap BF_{\left[a,b\right]}^{QH\backslash IH}\right) \end{array}$$

Let *n*_1_, *n*_2_, *n*_3_ and *n*_4_ denote the number of 1-bits in $\left|BF_{d^{\prime }}^{IH}\right|$, $\left|BF_{d^{\prime }}^{NH\backslash IH}\cap BF_{\left[a,b\right]}^{IH}\right|$, $\left|BF_{d^{\prime }}^{IH}\cap BF_{\left[a,b\right]}^{QH\backslash IH}\right|$ and $\left|BF_{d^{\prime }}^{NH\backslash IH}\cap BF_{\left[a,b\right]}^{QH\backslash IH}\right|$, respectively. For any hash functions *h_i_* and *h_j_* (*i* ≠ *j*), there is *h_i_*(*d′*) ≠ *h_j_*(*d′*). Hence, we have that: *n*_1_ = |*IH*|, 0 ≤ *n*_2_ ≤ min{|*IH*|, |*NH\IH*|}, 0 ≤ *n*_3_ ≤ min{|*IH*|, |*QH\IH*|} and 0 ≤ *n*_4_ ≤ min{|*NH\IH*|,|*QH\IH*|}. That is, *η_d_* ≥ |*IH*|, which contradicts the assumption. Therefore, *d′* ∉ [*a, b*], *i.e.*, if *η* < |*IH*|, there must be *d* ∉ [*a, b*].
